# The biomechanics and prevention of vein graft failure in coronary revascularization

**DOI:** 10.20517/2574-1209.2023.97

**Published:** 2023-12-14

**Authors:** Elbert E. Heng, Hanjay Wang, Oluwatomisin Obafemi, Alison Marsden, Y. Joseph Woo, Jack H. Boyd

**Affiliations:** 1 Department of Cardiothoracic Surgery, Stanford University School of Medicine, Stanford, CA 94305, USA.; 2 Stanford Cardiovascular Institute, Stanford University School of Medicine, Stanford, CA 94305, USA.; 3 Department of Bioengineering, Stanford University, Stanford, CA 94035, USA.

**Keywords:** Coronary artery bypass grafting, saphenous vein, vein graft failure, neointimal hyperplasia, external stenting, vein graft remodeling

## Abstract

Saphenous vein grafts (SVGs) are the most widely used conduit in coronary artery bypass grafting (CABG) surgery; however, SVG failures due to neointimal hyperplasia present a significant long-term limitation to the effectiveness of myocardial revascularization. This review will provide a comprehensive overview of the biological mechanisms of vein graft failure, including compensatory endothelial proliferation, extracellular matrix deposition, and adventitial disruption. We will discuss historical and emerging strategies for vein graft failure prevention with a focus on the role of mechanical vein graft support using external stenting. Finally, we will highlight the results of preclinical and human trials and discuss future directions for investigation.

## INTRODUCTION

Coronary artery bypass grafting (CABG) is the gold-standard treatment for increasing survival in patients with multivessel coronary artery disease (CAD), and saphenous vein grafts (SVGs) remain the most widely used conduit in over 350,000 surgeries performed annually in the United States^[1]^. Despite advances in surgical care and technique, SVG failure following CABG remains a significant cause of morbidity in the form of repeat revascularization, with failure rates approaching 50% at 10 years postoperatively^[2,3]^. The pathophysiology of vein graft failure is complex and multifactorial, involving the interplay between multiple biological and mechanical changes stemming from the transposition of vein grafts from low-pressure venous systems to pulsatile high-pressure arterial systems during bypass surgery. In particular, the constellation of early endothelial and smooth muscle cell proliferation, later extracellular matrix (ECM) deposition, and eventual atherosclerotic degeneration, collectively known as neointimal hyperplasia, has been identified as a therapeutic target in technologies aimed at preventing vein graft failure. Over the years, special attention has been given to extravascular stenting as a method of reducing neointimal hyperplasia by offloading vein graft wall stress, with several devices now being implemented in ongoing human clinical trials. In this review, we will aim to provide a comprehensive overview of the biology and prevention of SVG failure, with a focus on the role of external stenting. We will examine the underlying mechanisms of SVG failure, the current understanding of disturbed flow and its impact on graft biology, and the evidence supporting the use of external stenting as a preventive strategy. Additionally, we will discuss historical perspectives, modern approaches, and emerging strategies for studying SVG failure and its prevention in patients undergoing CABG.

## NATURAL HISTORY OF VEIN GRAFT FAILURE

Ever since Favorolo *et al*. pioneered its use in the 1960s, autologous saphenous vein has long been a conduit of choice in coronary bypass surgery due to its ready availability and ease of use^[4]^. SVGs are utilized in over 90% of patients undergoing CABG^[5]^, but they are associated with a high incidence of graft failure, which can, in turn, lead to adverse clinical outcomes. Historically, it has been estimated that 5%-10% of SVGs fail within 1 month of surgery, 10%-25% within 12–18 months, and 40%-50% within 10 years^[2,3,6]^. Various risk factors for SVG failure have been identified, including patient-related factors such as age, diabetes, and smoking, as well as surgical factors related to harvesting technique, graft storage solutions, and target coronary anatomy^[6]^.

Since the advent of CABG, the use of the left internal mammary artery (LIMA) has been recognized to confer significant prognostic benefit owing to its superior patency of up to 90% at 10 years^[2,7,8]^, particularly when associated with the left anterior descending (LAD) coronary artery territory. Likewise, multiple arterial grafting using radial artery (RA) and right internal mammary artery (RIMA) as second conduits has seen increasing adoption in the contemporary practice of coronary revascularization, supported by numerous studies consistently demonstrating significant improvements in event-free survival^[9,10,11]^. Nonetheless, total arterial grafting is not always feasible, and less than 10% of CABGs in North America utilize more than one arterial graft^[5,6]^. Therefore, SVG remains the most widely used conduit in CABG, with the prevention of SVG failure remaining a significant unmet clinical need.

The clinical significance of SVG failure in the setting of CABG is underscored by its impact on patient morbidity and mortality. Early studies, such as that by Lytle *et al*., reported significant mortality associated with SVG failure in the LAD territory, with only 50% survival at 5 years following catheterization demonstrating > 50% SVG stenosis^[12]^. In a large single-center cohort review of 1,388 patients undergoing CABG over a 25-year span, Fitzgibbon *et al*. reported that vein grafts to the LAD occluded early less often than grafts to other vessels; however, high profile lesions of > 50% stenosis were present even in 37% of all patent grafts by 12.5 years following surgery^[8]^. Analysis of data from the Duke Cardiovascular Databank by Halabi *et al*. has further corroborated the relationship between critical but non-occlusive SVG disease and adverse clinical outcomes of death, myocardial infarction, and repeat revascularization^[13]^. Because angiography in these early cases series was driven by the presence of clinical symptoms, it is likely they underestimated the true incidence of vein graft failure, and it is possible that vein graft failure accompanied by clinical symptoms is more prognostically important than vein graft stenosis alone. Since then, evolving CABG practices, including the routine use of LIMA instead of SVG to the LAD, have significantly improved survival after CABG. Indeed, in a contemporary study of 1,829 patients undergoing CABG by Lopes *et al*., 93% of grafts to the LAD were LIMA, and there was no association between SVG failure and death; however, there remained a significant association between SVG failure and the composite outcome of death, myocardial infarction, or revascularization, driven mainly by revascularization^[14]^.

## PATHOGENESIS OF VEIN GRAFT FAILURE

**Figure 1.**
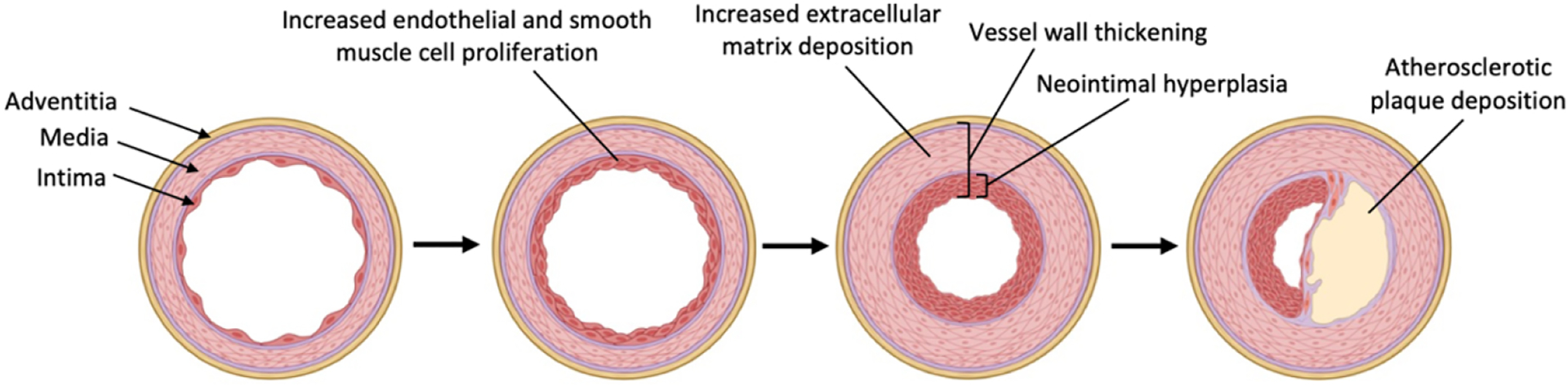
Biological cascade of neointimal hyperplasia in venous grafts. Created with BioRender.com.

SVG failure after CABG can manifest in either an early phase within one month of surgery, or in the intermediate to late phase spanning up to several years after surgery. While early vein graft failure is often attributed to technical aspects of surgery and resulting thrombosis, intermediate to late vein graft failure is predominantly driven by neointimal hyperplasia leading to progressive vessel wall thickening followed by superimposed atherosclerosis over time [Figure 1]. All SVGs are thought to be affected by neointimal hyperplasia to some degree, owing to the biological cascade triggered by exposure of thin-walled veins to the hemodynamic stresses of arterial circulation^[6]^. The pathophysiologic mechanisms of neointimal hyperplasia, therefore, serve as important therapeutic targets for the prevention of vein graft failure.

### Proliferative phase

The proliferative phase of neointimal hyperplasia is characterized by rapid endothelial cell (EC) and smooth muscle cell (SMC) proliferation in response to intimal injury. In native saphenous vein, the tunica intima comprises a confluent monolayer of endothelium and is surrounded by a tunica media layer consisting of an average of ten SMC layers^[15]^. Vein harvesting and grafting during bypass surgery produces a denuded area within the vessel wall of acute endothelium loss at anastomotic sites. Within the first 24 h after vein graft transposition, EC proliferation commences in order to re-establish an endothelial monolayer covering the denuded areas^[16,17]^. In rabbit models of jugular vein to carotid artery transposition, thymidine labeling indices indicate that EC proliferation increases 400-fold during the first week after vein grafting. By 2 weeks, denuded areas of vein grafts are completely reendothelialized, but EC proliferation continues at an increased rate until 12 weeks after the initial injury^[16,17]^.

Concurrently, during the first 48 h after vein transposition, platelets and leukocytes also adhere to denuded surfaces of vein grafts before the endothelial monolayer has been fully re-established. Platelet-derived growth factor (PDGF), released by these platelets, is thought to promote early SMC proliferation and migration into the intima. The peak activity of SMC proliferation occurs 1 week after surgery, and returns to near quiescent levels 4 weeks after surgery, after which SMC mass remains relatively constant as measured by morphometric analysis and DNA content. SMC thymidine labeling, however, can remain slightly elevated for up to 24 weeks after surgery^[16,17]^.

Following the initial response of endothelial and intimal regeneration, the cell proliferation phase of neointimal hyperplasia continues as a sustained process that extends over a considerable duration following surgery. In porcine models of saphenous vein to carotid bypass surgery, immunohistochemistry for proliferating cell nuclear antigen (PCNA) reveals heightened levels of cellular proliferation up to 6 months after surgery, with PCNA-positive cells being found most abundantly in the tunica media, suggesting broader involvement of multiple vessel layers in neointimal hyperplasia long-term^[18,19]^.

### Secretory phase

After cell proliferation levels peak during the initial month following vein graft transposition, the secretory phase of neointimal hyperplasia occurs, marked by increased production of extracellular matrix (ECM). The expanded SMC population from the proliferative phase of neointimal hyperplasia plays a vital role in this process, driving the deposition of ECM in the neointima and media^[15]^. In animal models of vein graft hyperplasia, tissue DNA content increases to approximately 5 times baseline during the first 4 weeks post-surgery, corresponding to rapid EC and SMC proliferation during this time. However, during weeks 4 to 24 after surgery, EC and SMC mass remain relatively constant, while DNA content per milligram of tissue drops by half, suggesting that continued wall thickening during this period is primarily a result of the deposition of non-cellular ECM consisting mostly of collagen and elastin, which serves to dilute the overall DNA content^[16]^. Vein graft wall thickening during the secretory phase reaches its maximum dimension at 12 weeks, along with vessel cross-sectional area^[17]^.

**Figure 2.**
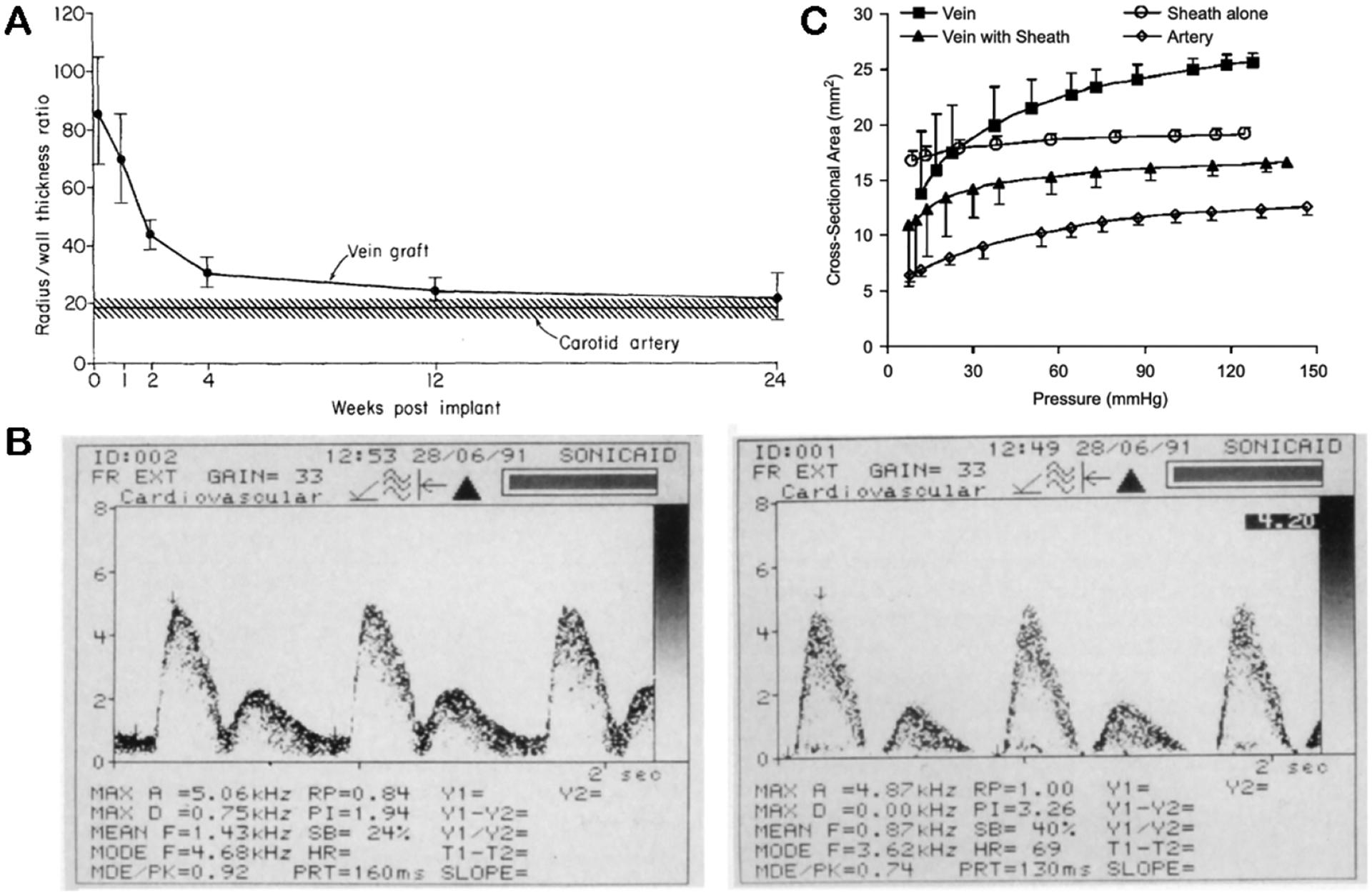
Biomechanical changes in vein grafts following transposition to arterial circulation. (A) Normalization of radius to wall thickness ratio over time in rabbit jugular vein to carotid interposition grafts. (B) Similarities in sonographic spectral flow patterns in unstented (left) and stented (right) porcine saphenous vein grafts. (C) Pressure to cross-sectional area ratio relationships in externally supported and non-supported saphenous veins from domestic hogs. Part A was adapted from Ref.^[16]^; part B was adapted from Ref.^[15]^; part C was adapted from Ref.^[21]^.

Biomechanical and hemodynamic factors, specifically tangential wall stress and shear stress, are thought to play an important role in determining the regulatory endpoints of vessel wall thickening in this secretory phase of neointimal hyperplasia. By the principle of Laplace’s Law, tangential wall stress of blood vessels is expressed in proportion to *P* × *r/t* of pressure *P*, radius *r*, and thickness *t*. Following the transposition of a thin-walled vein into a pulsatile and high-pressure arterial system, wall thickening is thought to be an adaptive response to lower the *r/t* ratio and normalize tangential wall stress in vein grafts. Indeed, in rabbit studies of jugular to carotid transposition, the *r/t* ratio of vein grafts was shown to return to values approximating the native carotid arteries by 12 weeks [Figure 2A], coinciding with the timepoint of maximum wall thickening in these models^[16]^. Likewise, wall shear stress, which is inversely proportional to cross-sectional area, is another key regulator of vein graft remodeling over time. Following the initial transposition of vein grafts into higher flow arterial circulation, it is theorized that veins will initially attempt to dilate beyond normal diameters in order to compensate against higher shear stress, but likely have lower set points as compared to arterial vessels^[17]^. Resulting areas of lower and oscillating shear stress in pulsatile circulation from this adaptation may, in turn, serve as niduses for continued intimal proliferation and eventual atherosclerotic plaque formation.

## BIOLOGY OF EXTERNAL VEIN GRAFT STENTING

Developing methods and strategies to prevent vein graft failure has been the subject of significant research and investigation. While medical therapies such as anti-platelet agents and lipid-lowering drugs have been shown to improve early patency of SVGs, the prevention of long-term failure related to neointimal hyperplasia remains a significant unmet need. Recognizing the importance of biomechanical stresses on vein graft thickening, the use of synthetic external supports has garnered significant interest as a surgical solution to protect vein grafts and promote favorable remodeling. External stent design aspects, including stiffness, sizing, porosity, and biodegradability, all play a critical role in consequent biological alterations to reduce neointimal hyperplasia and enhance long-term patency. Depending on individual stent properties, the mechanism by which they decrease neointimal hyperplasia can involve one or several biological processes.

### Hemodynamic modulation

One of the first descriptions of external vein graft support was introduced by Parsonnet *et al*. in the 1960s, in which tubular scaffolds were applied to the jugular veins of dogs undergoing jugular to carotid interposition surgery^[20]^. This early sheath model was constructed with knitted monofilament fibers of polyethylene, polypropylene, and Teflon, woven into a fabric capable of adjusting transverse diameter by changing longitudinal tension, akin to a child’s finger trap. Initially, the rationale behind this stent design was aimed at reducing the size disparity between smaller arteries and larger vein grafts, which was believed to be a cause of turbulent flow and thrombosis. While the adjustable sheath successfully prevented overdistention of vein grafts, later Doppler studies demonstrated similar flow rate and flow uniformity (turbulence) profiles between stented and non-stented vein grafts at the carotid position^[15]^ [Figure 2B]. Subsequent investigations of vein graft support expanded their scope to explore hemodynamic parameters such as tangential wall and shear stress with an aim towards the prevention of neointimal hyperplasia rather than thrombosis.

In a seminal study by Kohler *et al*., polytetrafluoroethylene (PTFE) sheaths were applied to the proximal segment of rabbit jugular vein to carotid artery interposition grafts, with the distal non-wrapped segment serving as control^[17]^. At 12 weeks, vessel wall hypertrophy was significantly less in tight-fit wraps as compared to looser-fit wraps and unwrapped controls, supporting the hypothesis that the degree of wall stress reduction as determined by the tightness of fit plays a role in attenuating wall thickening. However, despite reduced wall stress, flow separation was still observed between wrapped and unwrapped vein segments, with Doppler measurements demonstrating high central velocities and occasional flow reversal near the vessel walls. Due to concerns that proximal segment wrapping likely impacted distal flow dynamics in this experimental paradigm, subsequent studies extended wrapping vein grafts along their entire length.

Enclosing vein grafts with rigid external supports theoretically has the potential to reduce or eliminate wall tension entirely. However, by limiting vessel expansion, the resulting mean shear stress may become higher than in unstented grafts. In a study by Liao *et al*., *ex-vivo* pressurization of saphenous veins when supported by poly-lactic-co-glycolic acid (PLGA) demonstrated that veins supported with external sheaths exhibited pressure to cross-sectional area (CSA) relationships closely approximating that of the sheath alone, resulting in a stress-strain curve intermediating between those of unsupported veins and those of native arteries [Figure 2C]^[21]^. With serial pressure testing of biodegradable PLGA sheaths, it was found that circumferential stress and modulus gradually increased over a period of 12 days, corresponding to PLGA degradation. Despite providing only partial and temporary mechanical support, biodegradable sheaths in this study still resulted in a reduction in vein graft wall thickness by 12 days. These data, together with later studies exploring remodeling benefits associated with oversized external stents, suggest that the therapeutic mechanism of external vein graft supports likely involves a combination of biological factors beyond hemodynamic modulation alone.

### Vessel wall morphology

While many studies of external vein graft support generally report some degree of reduction in wall thickening, the remodeling seen in distinct vessel wall layers may vary depending on the properties of the particular stent applied. Overall, the biological response of vein grafts to external stenting is multifaceted and involves changes in both matrix deposition and cell proliferation.

#### Medial hypertrophy

Across multiple sheath iterations in literature, external vein graft stenting is consistently reported to reduce the thickening of the tunica media layer of grafted veins. In studies of PTFE sheaths applied to SVGs in porcine carotid bypass surgery, the tunica media CSA was found to be twice as thick in unstented *vs*. stented vein grafts. Additionally, while the internal elastic lamina remained unchanged at 4 weeks, the external elastic lamina became more separated as a result of this hypertrophy^[18]^. Although both stented and unstented SVGs demonstrated increased matrix deposition in comparison to ungrafted saphenous veins, cell densities did not differ significantly between the two groups at 1 and 6 months, suggesting that reduced cell proliferation rather than decreased matrix deposition was responsible for the observed reductions in medial hypertrophy. Supporting this hypothesis, PTFE-stented vein grafts have demonstrated absent PCNA expression in the media alongside an increased presence of infiltrating microvessels, whereas unstented vein grafts exhibited abundant PCNA expression with absent microvessels. Growth factor activity, particularly that of PDGF, also appears to play a substantial role in the reduction of medial cell proliferation. In a study by Mehta *et al*., they found widespread distribution of PDGF α and β receptor subunits in all vessel layers of both stented and unstented SVGs, and observed decreased expression of specifically the PDGF-BB isoform in stented vein grafts, with PDGF-AA expression remaining similar between two groups^[19]^. Together, these findings emphasize the importance of growth factor signaling and chemotaxis tunica media remodeling following external vein graft stenting.

#### Neointimal thickening

Unlike the consistent reductions seen with medial thickening, studies of external vein graft stenting have demonstrated mixed effects on the tunica intima. Although denuded endothelium at vein graft anastomotic sites is fully restored within 2 weeks of grafting, cell proliferation continues to form a neointimal layer consisting of axially and circumferentially oriented SMCs. In a study by Violaris *et al*., they reported that external stenting with PTFE produced a two-fold increase in the intimal CSA of saphenous veins, essentially neutralizing the concurrent reduction in medial thickness^[15]^. Consequently, although the overall impact of PTFE stenting on wall thickness was negligible, luminal encroachment was increased by stent grafts due to restriction of the outer vessel diameter. In an effort to reduce luminal encroachment, subsequent research explored the use of oversized and non-restrictive stents. In a study by Izzat *et al*., they examined the application of 5-, 6-, and 8-mm Dacron velour stents to saphenous veins in carotid interposition surgery^[18]^. At 4 weeks, all stented vein grafts demonstrated equal reductions in medial thickness, but only 6- and 8-mm stented vein grafts showed significant reductions in neointimal thickness in contrast to 5-mm stented grafts with comparatively greater intimal thicknesses. At odds with early stent designs advocating tight-fit and diameter reduction of vein grafts, these data and subsequent work have proposed that oversized loose-fit vein graft supports produce more effective suppression of intimal thickening^[19,22,23]^. Additionally, the microporous nature of stent materials such as Dacron velour is thought to play a role, in contrast to microporous PTFE used previously.

#### Neoadventitial formation

Apart from their influence on vessel intima and media, external vein graft stents have been shown to produce cellular and chemotactic changes to the adventitial environment surrounding vein grafts which can contribute to either favorable or unfavorable remodeling. In early studies of tight-fitting stents, rigid sheaths surrounding vein grafts would produce a foreign body giant cell reaction and become enveloped completely by fibrous tissue over time^[20]^. In later studies utilizing more porous and less-constrictive stents, the space between vein grafts and external stents was observed to become organized into a neoadventitia containing macrophages, giant cells, and new microvessel growth^[22]^. Within this neoadventitia, it was theorized that accumulated macrophages were responsible for enhancing microvessel formation by releasing proangiogenic cytokines, which would consequently reduce graft wall hypoxia to create a favorable chemotactic gradient encouraging the migration of SMCs outwards and away from the intima. Indeed, while PDGF and PCNA expression are decreased or absent from the media of stented vein grafts, high densities of PCNA-positive nuclei and detectible PDGF have been observed in the neoadventitia, indicating ongoing perivascular cell proliferation^[19]^. Likewise, in vein grafts fitted with constrictive supports, it is possible that the tighter-fitting sheaths disrupted neoadventitial microvascularity, explaining why they produced paradoxical increases in intimal thickness. The dynamic interplay between stent materials, dimensions, and neoadventitial formation highlights the intricate nature of vein graft remodeling, and has served to inform modern-day strategies to prevent vein graft failure.

## MODERN ADVANCEMENTS IN VEIN GRAFT FAILURE

The contemporary landscape of vein graft failure prevention has been marked by significant advancements in the understanding of its pathology, which have in turn informed the development and optimization of novel treatment strategies. In particular, the integration of computational modeling and molecular gene expression profiles in vein grafts has yielded new insights into the complex interaction between hemodynamic perturbations and cellular signaling. Concurrently, advancements in biomaterials engineering and fabrication techniques have also produced novel external support compositions with tunable biodegradability profiles, some of which are now being applied in ongoing human trials.

### Computational modeling of vein graft adaptation

In modern studies of vein graft failure, computation fluid dynamics (CFD) has emerged as a valuable method for studying the complex flow environments of saphenous veins in coronary surgery. Whereas historical studies of vein graft flow have relied on simplified assumptions and idealized vessel geometries, CFD models are able to incorporate patient-specific anatomy to investigate differential and oscillating distributions of hemodynamic stresses within CABG conduits. Various hemodynamic parameters have been developed to characterize the diverse flow environments between stented and unstented vein grafts, including time-averaged wall shear stress (TAWSS) reflecting the frictional force of blood flow on the endothelium, oscillatory shear index (OSI) representing the magnitude and direction oscillation of wall shear stress^[24]^, and relative residence time (RRT) indicating relative time duration that blood elements remain at the endothelium^[25]^. CFD analysis of these parameters using human patient data from the venous external support trial (VEST) has revealed distinct flow patterns associated with external support devices^[26–28]^. While TAWSS (determined by mean diameter and flow rate) was shown to be expectedly similar between stented and unstented vein grafts, unstented SVGs demonstrated high OSI values correlating with the development of diffuse intimal hyperplasia^[27]^. Depending on overall graft topography, OSI can differ significantly between grafts with equivalent TAWSS, and thus, the modern understanding of vein graft remodeling has expanded to emphasize the importance of improving overall graft geometry, rather than diameter modulation alone.

Beyond hemodynamic insights, computational methodologies have also been applied to the development of predictive models to simulate the adaptive capacity of veins and identify mechanisms of maladaptation in bypass grafts. In 2015, Ramachandra *et al*. introduced a growth and remodeling (G&R) framework of vein graft adaptation using constrained mixture theory to integrate existing knowledge of fluid dynamics with cell-mediated responses of vascular wall elements (collagen, elastin, and SMCs)^[29]^. In these cell-mediated G&R models, simulated veins were subjected to a range of combined pressure and flow in arterial circulation to identify the upper and lower bounds of vein adaptability. Repeated computational testing with this model has suggested that gradual mechanical loading over 8 days rather than abrupt change seen in clinical practice greatly increased the adaptive capacity of veins^[30]^, a finding that has inspired biodegradable designs of modern vein graft sheaths^[31]^.

### Molecular gene expression in vein graft remodeling

While changes in EC and SMC proliferation along with matrix deposition have long been appreciated in vein graft remodeling, studies of molecular gene expression in grafted veins have uncovered specific proteins and chemical pathways driving these pathologic adaptations. In response to arterial pressurization, vein graft SMCs have been shown to undergo loss of differentiation via downregulation of the Notch1/Ephrin-B2 (arterial marker) and Notch4/Ephrin-B4 (venous marker) pathways, and additionally switch from a contractile to synthetic phenotype with markedly decreased SM22α and calponin expression^[32]^. Endothelial dysfunction also ensues with persistent downregulation of endothelial nitric oxide synthase (eNOS). Following alterations in cell proliferation, matrix metalloproteinases (MMPs) and tissue inhibitors of these enzymes (TIMPs) have also been shown to drive ECM remodeling, with upregulation of MMP-2, MMP-9, TIMP-1, and TIMP-2. In comparison to unsupported vein grafts, external stenting appears to attenuate ECM remodeling and fibrosis by counteracting the upregulation of plasminogen activator inhibitor-1 (PAI-1) and transforming growth factor beta II (TGFβ2). Additionally, heatmaps of differential gene expression between wrapped and non-wrapped vein grafts have also identified downregulation of collagen 11A1 (COL11A1) and thrombospondin-4 (THBS4) as atheroprotective mechanisms of external supports, as well as decreased fibrillin 2 (FBN2) and LIM domain only 7 (LMO7) corresponding to decreases in maximum wall thickness^[31]^.

### Modern therapies to prevent vein graft failure

**Figure 3.**
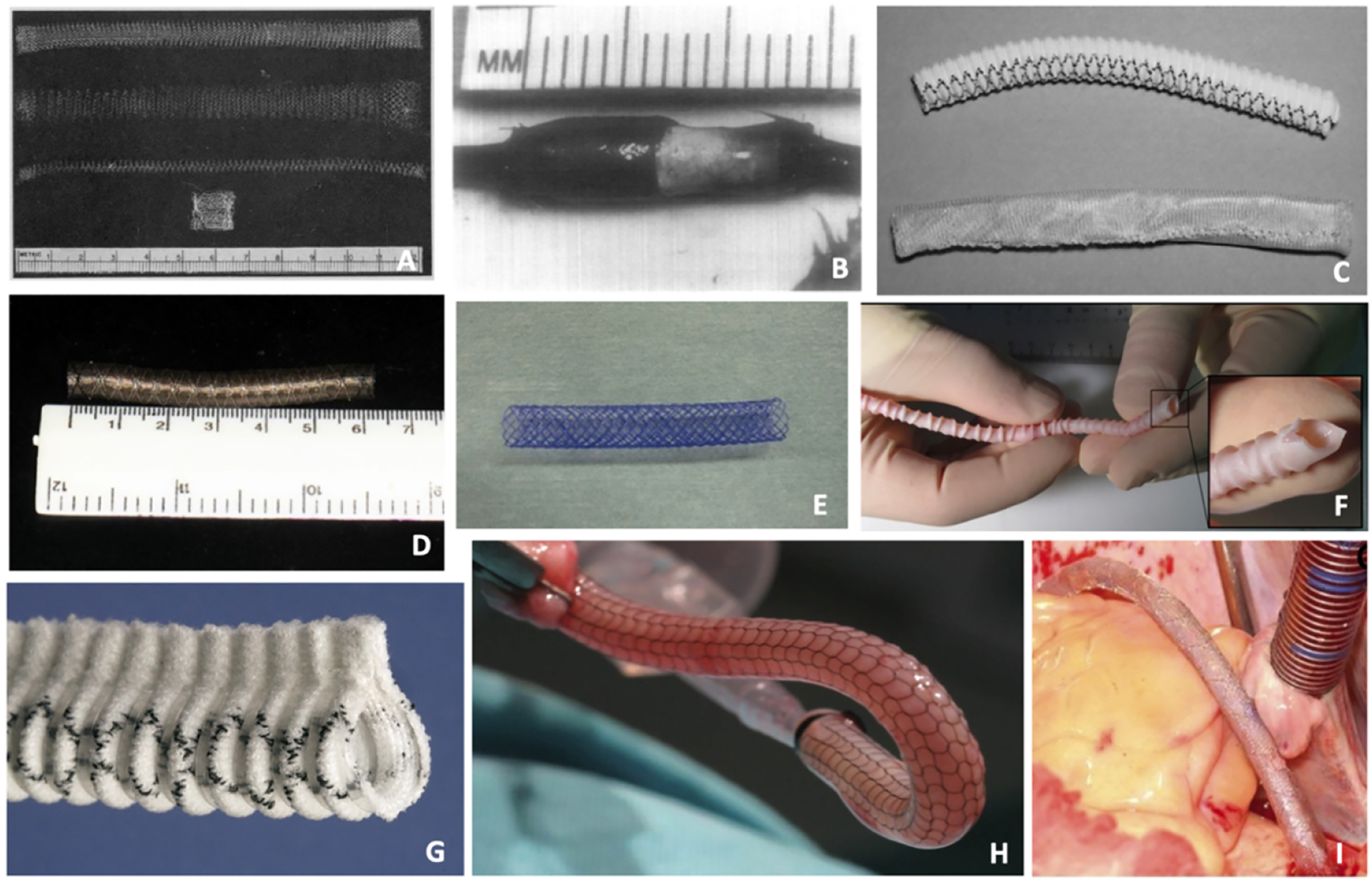
Evolution of external vein graft stent design. (A) Knitted fabric stent of polyethylene, polypropylene, and Teflon^[20]^. (B) Polytetrafluoroethylene (PTFE) sheath applied to proximal rabbit jugular vein graft^[17]^. (C) Loose-fitting external polyester stent (above) and polyglactin (Vicryl) sheath (below)^[22]^. (D) Expandable braided cobalt-chromium alloy stent^[33]^. (E) Poly L-lactide-ε-caprolactone (75/25) copolymer (P (LA/CL)) mesh^[34]^. (F) Electrospun poly(ester urethane)urea (PEUU) stent^[36]^. (G) Extent (Vascutek Ltd, Inchinnan, Scotland) external Dacron stent^[47]^. (H) Knitted nitinol eSVS mesh (Kips Bay Medical Inc, Minneapolis, MN, USA)^[48]^. (I) Braided cobalt-chromium VEST (Vascular Graft Solutions Ltd, Tel Aviv, Israel)^[50]^. Photographs adapted from respective references.

Contemporary iterations of external vein graft stents have leveraged advancements in biomaterials engineering and fabrication techniques to build on historical techniques and evolving knowledge of vein graft remodeling mechanisms [Figure 3]. Reminiscent of the “finger trap” concept first proposed in seminal work by Parsonnet *et al*., recent expandable stent devices have been made using braided cobalt-chromium alloy fibers in order to produce kink-resistant external supports capable of conforming to a variety of vessel diameters^[20,33]^. Likewise, efforts to minimize long-term foreign body reactions from non-degradable stents have led to the exploration of biodegradable materials including poly L-lactide- ε-caprolactone (75/25) copolymer (P (LA/CL)) mesh^[34]^ and poly(ester urethane)urea (PEUU)^[35]^, the latter of which has been electrospun directly onto sheep saphenous veins to produce patient-specific external graft support^[36]^.

Finally, woven mesh materials including soft polyester, Monocryl, and Vicryl have been fashioned into “appropriate fit” external supports that closely appose vessel walls while still allowing neoadventitial vascularization between mesh fibers^[22,32,37]^. Together, these innovations in external stent design and production aim to overcome previous limitations of early stent devices and enhance long-term vein graft patency in CABG.

In light of increasing awareness of the molecular signaling pathways contributing to vein graft failure, researchers have also explored peri-adventitial treatments targeting these pathways as an alternative to external stent usage. For instance, periadventitial applications of elastomer gels containing diethylenetriamine nitric oxide (NO) adduct have been explored as a way to suppress platelet aggregation and restore endothelial dysfunction from downregulation of eNOS after vein grafting^[38]^. Adventitial administration of gene transfer vectors and nanoparticles have also been studied to deliver platelet-derived endothelial cell growth factor (PD-ECGF) thymidine phosphorylase (TP)^[39]^ and microRNA-145 (miR-145)-loaded poly(lactic-co-glycolic acid) (PLGA)^[40]^ to modulate SMC chemotaxis and phenotype from proliferative to contractile states. Fibrin sealants such as cyanoacrylate^[41,42]^, anti-adhesive agents such as hyaluronic acid-carboxymethyl cellulose (HA/CMC)^[43]^, and collagen cross-linking with photoactive tissue passivation^[44]^ have also been investigated as methods of providing mechanical offloading to vein grafts without sheath implants. As with external vein graft stents, the long-term effects of these adventitial treatments remain to be seen and will remain a subject of future investigation.

## HUMAN CLINICAL TRIALS OF VEIN GRAFT FAILURE PREVENTION

Over the years, several therapies demonstrating promising preclinical data for the prevention of vein graft failure have been implemented in clinical trials. However, translation from laboratory to human application has produced mixed results, with some treatments demonstrating encouraging findings and others falling short of anticipated outcomes.

### PREVENT IV

The Project of *Ex-vivo* Vein Graft Engineering via transfection (PREVENT IV) trial was a phase III, multicenter, randomized, double-blind, placebo-controlled trial that evaluated the efficacy and safety of edifoligide, an E2F transcription factor decoy shown to reduce intimal hyperplasia in animal models, in preventing vein graft failure following CABG^[45]^. A total of 3,014 patients undergoing CABG with at least two planned vein grafts were enrolled. The primary endpoint was the rate of vein graft failure at 12–18 months after enrollment. Both at 1- and 5-year follow-ups, edifoligide was found to be no more effective than placebo in preventing vein graft failure, and did not affect outcomes or survival after CABG^[45,46]^.

#### Extent study

The Extent study was a phase I randomized clinical trial that enrolled 20 patients undergoing isolated CABG to evaluate the use of an external Dacron stent for the prevention of vein graft failure^[47]^. The Extent (Vascutek Ltd, Inchinnan, Scotland) was composed of an incomplete tube of macroporous knitted polyester reinforced with PTFE ribs at 1 cm intervals to form a flange. On follow-up angiography between 6–18 months after CABG, 17 of 20 Extent grafts were thrombosed, while all LIMA and non-extent vein grafts remained patent. The disappointing universal thrombosis of Extent vein grafts in this study was attributed to stent rigidity and graft kinking resulting from the flanged edge design.

#### eSVS Mesh

The eSVS mesh (Kips Bay Medical Inc, Minneapolis, MN, USA) was an external graft stent comprised of a knitted Nitinol mesh tube, and was advertised as being highly flexible and kink resistant. The procedure of eSVS application in CABG involved selecting an appropriately sized device based on saphenous vein measurement, pulling the SVG through the mesh tube, and finally fixing the mesh to the vein using fibrin glue. As part of a prospective, randomized, multicenter trial, Schoettler *et al*. reported that among 25 subjects enrolled in their clinic, a nine-month follow-up demonstrated 27.8% patency of mesh-supported grafts *vs*. 85.7% patency in conventional vein grafts^[48]^. Inderbitzin *et al*. similarly reported 76% patency of meshed SVGs at 1 year *vs*. 100% patency in non-meshed SVGs. Inferior patency in these studies was believed to be related to the use of fibrin glue and stent undersizing^[49]^.

### VEST

Perhaps the most prominently studied in-human device for the prevention of vein graft failure has been the VEST (Vascular Graft Solutions Ltd, Tel Aviv, Israel). The VEST device is an external sheath composed of braided cobalt-chromium alloy that had demonstrated promise in animal models of CABG, and has since been investigated in multiple randomized controlled trials. In the VEST I trial, 30 patients undergoing CABG were enrolled to receive one external stent device to a single SVG randomly assigned to either the right or circumflex coronary territory^[50]^. At 1-year follow-up angiography, the primary endpoint of intimal hyperplasia by mean wall area was significantly reduced (4.37 ± 1.40 mm^2^ *vs*. 5.12 ± 1.35 mm^2^, *P* = 0.04) with VEST application, while early graft failure was not significantly different between stented and non-stented vein grafts (30% *vs*. 28.2%, *P* = 0.55). Additionally, SVG failure rates in both groups were noted to be significantly lower in the left circumflex (17.6% *vs*. 27.5%, *P* = 0.02) as compared to the right coronary territory (46.2% *vs*. 13.4%, *P* = 0.01), and this was suspected to be related to the use of metallic clips to ligate SVG side branches and the use of suture in some cases to secure the study device to the graft. With these findings in mind, the VEST II trial was designed to investigate if avoiding suture fixation and metal clips could improve the early patency of externally stented SVGs in the right coronary territory^[51]^. In total, 30 patients undergoing CABG were assigned to have external stenting of an SVG to the right coronary artery specifically, and were found to have an improvement of graft patency to 86.2% during the 3 to 6-month follow-up. Long-term outcomes of 21 patients from the original VEST I trial were reported in the extended follow-up VEST IV study^[52]^. While vein graft failure rates did not differ significantly at 4.5 years between stented and control grafts (30% *vs*. 23%, *P* = 0.42), Fitzgibbon grade I patency reflecting no luminal irregularities was higher in stented than non-stented grafts (81% *vs*. 48%, *P* = 0.42), suggesting improved remodeling associated with external stenting. Extended outcomes from a larger cohort of 184 patients undergoing CABG with the VEST sheath have also been reported in the VEST III European multicenter randomized within-patient control trial^[53]^. Consistent with the findings of VEST IV, 2-year follow-up angiography and intravascular ultrasound demonstrated similar overall patency between groups (78.3% *vs*. 82.2%, *P* = 0.43), and improved Fitzgibbon patency scale I rate (66.7% *vs*. 54.9%, *P* = 0.03). In the USA, 224 patients have been recruited into the VEST Pivotal trial examining study endpoints of graft patency and intimal hyperplasia at 1 year, as well as major adverse cardiac and cerebrovascular events annually over 5 years. Though early graft patency did not differ significantly with the VEST, potential long-term benefits associated with improved luminal geometry in patent vein grafts are yet to be seen^[54]^.

## CONCLUSION

The study and prevention of vein graft failure after coronary bypass surgery has seen significant advancements that have paved the way for the development of novel devices and treatment strategies. While existing therapies translated to human application have not yet demonstrated clinical benefit, long-term follow-up and continued investigation in these trials are needed. With continued innovation in biomaterials and fabrication technologies, next-generation devices of external vein graft support hold promise for providing patient-specific and targeted biological solutions to reduce neointimal hyperplasia and achieve long-term vein graft patency. Integrating these advancements into future translational efforts will be pivotal in enhancing current approaches to prevent vein graft failure, and ultimately improve patient outcomes after CABG.
